# Structural and functional divergence of two fish aquaporin-1 water channels following teleost-specific gene duplication

**DOI:** 10.1186/1471-2148-8-259

**Published:** 2008-09-23

**Authors:** Angèle Tingaud-Sequeira, François Chauvigné, Mercedes Fabra, Juanjo Lozano, Demetrio Raldúa, Joan Cerdà

**Affiliations:** 1 Laboratory of Institut de Recerca i Tecnologia Agroalimentàries (IRTA)-Institut de Ciències del Mar, Consejo Superior de Investigaciones Científicas (CSIC), 08003 Barcelona, Spain; 2 Centro de Investigación Biomédica en Red de Enfermedades Hepáticas y Digestivas (CIBERehd), 08036 Barcelona, Spain; 3 Génomique et Physiologie des Poissons, Université Bordeaux 1, UMR NuAGe, 33405 Talence, France; 4 Omnia Molecular, Barcelona Science Park, 08028 Barcelona, Spain; 5 Laboratory of Environmental Toxicology, Universidad Politécnica de Catalunya, 08220 Terrassa, Spain

## Abstract

**Background:**

Teleost radiation in the oceans required specific physiological adaptations in eggs and early embryos to survive in the hyper-osmotic seawater. Investigating the evolution of aquaporins (AQPs) in these vertebrates should help to elucidate how mechanisms for water homeostasis evolved. The marine teleost gilthead sea bream (*Sparus aurata*) has a mammalian aquaporin-1 (AQP1)-related channel, termed AQP1o, with a specialized physiological role in mediating egg hydration. However, teleosts have an additional AQP isoform structurally more similar to AQP1, though its relationship with AQP1o is unclear.

**Results:**

By using phylogenetic and genomic analyses we show here that teleosts, unlike tetrapods, have two closely linked AQP1 paralogous genes, termed *aqp1a *and *aqp1b *(formerly AQP1o). In marine teleosts that produce hydrated eggs, *aqp1b *is highly expressed in the ovary, whereas in freshwater species that produce non-hydrated eggs, *aqp1b *has a completely different expression pattern or is not found in the genome. Both Aqp1a and Aqp1b are functional water-selective channels when expressed in *Xenopus laevis *oocytes. However, expression of chimeric and mutated proteins in oocytes revealed that the sea bream Aqp1b C-terminus, unlike that of Aqp1a, contains specific residues involved in the control of Aqp1b intracellular trafficking through phosphorylation-independent and -dependent mechanisms.

**Conclusion:**

We propose that 1) Aqp1a and Aqp1b are encoded by distinct genes that probably originated specifically in the teleost lineage by duplication of a common ancestor soon after divergence from tetrapods, 2) Aqp1b possibly represents a neofunctionalized AQP adapted to oocytes of marine and catadromous teleosts, thereby contributing to a water reservoir in eggs and early embryos that increases their survival in the ocean, and 3) Aqp1b independently acquired regulatory domains in the cytoplasmatic C-terminal tail for the specific control of Aqp1b expression in the plasma membrane.

## Background

Membrane intrinsic proteins (MIP) such as aquaporins (AQP) are molecular channels present from bacteria to humans that transport water and small molecular weight solutes across biological membranes [1]. These membrane proteins are classified into two groups: those that are water-selective, and those that also transport small neutral molecules, such as glycerol and urea, termed aqua(glycero)porins. All known AQPs have six transmembrane domains connected by five loops (A-E), in which both the N- and C-termini are cytoplasmic. Their primary structure can be divided into two similar halves each of which bear the highly conserved Asn-Pro-Ala (NPA) motif in loops B and E that are involved in the formation of the water pore, which is the hallmark of the MIP family [1,2]. In higher vertebrates, 13 different AQPs have been described, which differ in tissue expression, regulation and selectivity [1,3].

Recent studies have shown that, in mammals, AQP1 and AQP2 are essential for water resorption in the kidney [4], AQP4 is involved in cerebral water balance, astrocyte migration and neural signal transduction [5], and AQP3 and AQP7 seem to play important roles during skin hydration and metabolism of adipocytes, respectively [6]. However, there is little information on the functional properties and physiological functions of AQPs in teleosts, vertebrates which form a highly diverse group of organisms adapted to living both in freshwater and seawater.

Marine teleosts are thought to have colonized the oceans after a long freshwater ancestry, which is supported by the fossil record and by the hypo-osmotic condition and the presence of a glomerular kidney in extant marine species (see [7] for review). The re-entry of teleosts into seawater, however, most likely required new molecular adaptations to maintain water homeostasis, which is especially important for gametes and early embryos that do not have osmoregulatory systems. The spawning of pelagic eggs by many marine teleosts (termed pelagophils), where water content may reach up to 95% in weight, has been proposed as a mechanism to provide a water reservoir in the embryo to compensate for the passive water efflux due to the hyper-osmotic effect of the seawater until osmoregulatory organs develop [7,8]. In addition, hydration of the egg contributes to buoyancy, thereby improving oxygen exchange and dispersal in the ocean.

Egg hydration in marine fish occurs during the later stages of oogenesis, prior to ovulation (i.e., oocyte meiotic maturation). It is driven by the osmotic gradient created by the generation of a large pool of free amino acids (FAAs) in the oocyte, produced from the hydrolysis of vitellogenin (Vtg)-derived yolk proteins, and the accumulation of inorganic ions (see [9] for review]. In the pelagophil teleost gilthead sea bream (*Sparus aurata*), we recently showed that water influx into the oocyte is facilitated by a novel water-selective AQP, predominantly expressed in the ovary, which, being structurally and functionally similar to mammalian AQP1, was named the *S. aurata *aquaporin-1 of the ovary (SaAQP1o) [10,11]. This finding illustrates how marine teleosts have evolved novel molecular mechanisms to face life in the ocean. However, sea bream also expresses another water-selective, AQP1-related channel, termed SaAQP1, which is more similar to mammalian AQP1 and is ubiquitiously distributed in tissues, including osmoregulatory organs such as the kidney, gills and intestine [10,12]. Water channels related to SaAQP1o and SaAQP1 have also been found in other teleosts [13-15], but the phylogenetic and functional relationships between these two vertebrate AQPs remain unclear.

Now that the genome of several teleosts has been completely or partially sequenced, and there is an increasing number of expressed sequence tags (ESTs) and cDNAs available, the phylogeny and functional properties of teleost AQP1-like proteins can be investigated. Using phylogenetic reconstruction and genomic analysis, we show here that the AQP1o gene (*aqp1b*) is teleost specific and probably originated by local duplication of a vertebrate AQP1 ancestor, while tetrapods have only a single *AQP1 *gene structurally more similar to teleost AQP1 (*aqp1a*). Expression analyses and functional characterization in *Xenopus laevis *oocytes suggest that teleost Aqp1b represents a neofunctionalized water channel in the ovary and some osmoregulatory organs of marine species, which has evolved unique regulatory domains at the C-terminal cytoplasmic tail for the control of intracellular trafficking.

## Results

### Duplication of AQP1 in teleosts

**Figure 1:**
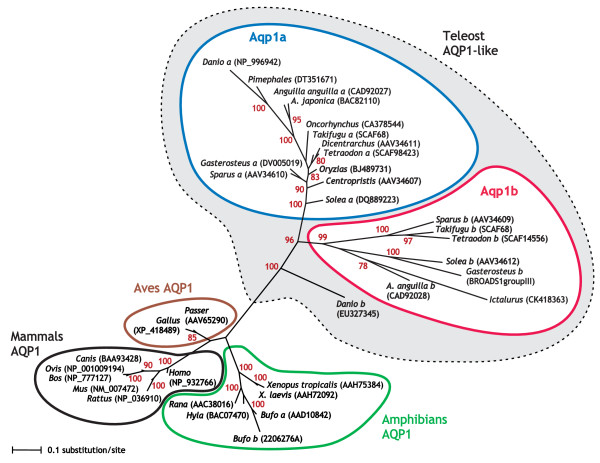
**Phylogenetic relationships of AQP1-like proteins in vertebrates**. Bayesian majority rule consensus phylogenetic tree for the amino acid alignment of teleost and tetrapod AQP1-like sequences. Nodes with ≥70% Bayesian posterior probabilities are shown. Branch lengths are proportional to BI estimates of numbers of amino acid substitutions. The GenBank accession number, scaffold, or chromosome group are indicated in parenthesis for each sequence.

AQP1-related, non-redundant amino acid sequences of teleosts were obtained by searching available genome and protein databases, and by cDNA cloning. The teleost sequences were aligned with the amino acid sequence of tetrapod AQP1 and the phylogenetic analyses conducted using the neighbour-joining (NJ), maximum likelihood (ML), and Bayesian inference (BI) methods (Figure 1; see also Additional file 1). The phylogenetic tree obtained consistently separated tetrapod AQP1 from the teleost lineage (NJ: 100; ML: 100; BI: 100), which appeared to have undergone a duplication event giving rise to the Aqp1a and Aqp1b subgroups (NJ: 92; ML: 46; BI: 96). The length of the branches of Aqp1b sequences were in general longer than those of Aqp1a, specially that of the zebrafish Aqp1b, suggesting a higher rate of residue mutations than members of the parent group, and hence a rapid evolution. Zebrafish (*Danio rerio*) Aqp1b, however, clustered significantly outside the Aqp1a and Aqp1b subgroups, which may be a result of the well-known long-branch attraction effect that can pull long branches towards the outgroup (in this case the tetrapods). In the amphibian *Bufo marinus*, two AQP1 sequences were found, termed *Bufo *a and *Bufo *b, that clustered together with the other amphibian AQP1, confirming that neither was a homolog of the teleost Aqp1b subgroup. These results confirmed the presence of two paralogous gene copies early in the Actinopterygii (Teleostei) lineage, related to human AQP1, that may have arisen after an early duplication event of an ancestral AQP1 gene. Thus, the two paralogous genes were termed *aqp1a *and *aqp1b *according to the nomenclature established for zebrafish [16]. The cDNAs encoding sea bream SaAQP1 and SaAQP1o, and Senegalese sole (*Solea senegalensis*) AQP1o, previously isolated [10], correspond to the transcripts of *aqp1a *and *aqp1b *paralogs, respectively. The European eel (*Anguilla anguilla*) Aqp1b cDNA that we cloned here is 100% identical to that recently isolated from the same species and named AQP1dup [14].

### Genomic organization of teleost *aqp1 *and *aqp1b *and primary structure of the encoded polypeptides

**Figure 2:**
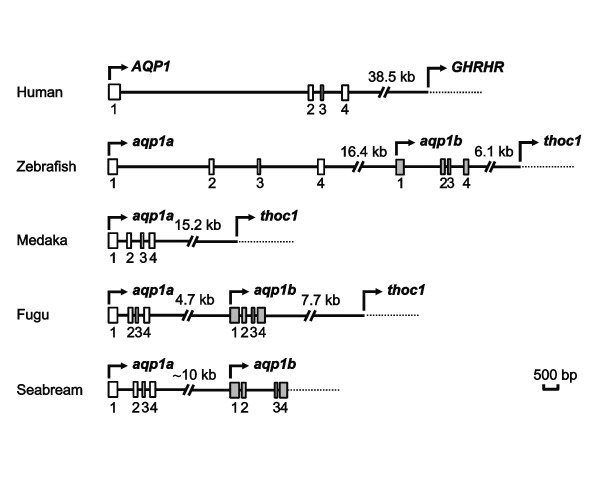
**Genomic organization of human AQP1 and teleost *aqp1a *and *aqp1b *genes**. Schematic representation of human AQP1 [67], and zebrafish, fugu, medaka and sea bream *aqp1a *and *aqp1b *gene loci. White (human AQP1 and teleost *aqp1a*) and grey (teleost *aqp1b*) boxes indicate exons with coding regions only. Downstream genes from human *AQP1* and teleost *aqp1b* are growth hormone releasing hormone receptor (*GHRHR*) and THO complex subunit 1 (*thoc1*), respectively.

In the genome of pufferfish (*Tetraodon nigroviridis*), zebrafish, stickleback (*Gasterosteus aculeatus*) and fugu (*Takifugu rubripes*), *aqp1a *and *aqp1b *are located in the same chromosome (15, 2, III, and scaffold 68, respectively). In all these species, both chromosome loci were found to be linked, *aqp1b *being downstream of *aqp1a*. Based on this synteny, the corresponding genomic region in sea bream was obtained by PCR, employing cDNA-based oligonucleotides, and sequenced, to compare the genomic organization of *aqp1a *and *aqp1b *between extant and more primitive teleosts (Figure 2). In zebrafish, *aqp1a *and *aqp1b *were separated by 16.4 kb and were 11.6 kb and 3.6 kb in length, respectively, whereas in sea bream both loci were separated by approximately 10 kb and were 2.1 kb and 2.8 kb in length, respectively. In fugu and *Tetraodon*, which have more compact genomes, *aqp1a *and *aqp1b *were 1.4–1.5 kb and 1.1–1.2 kb in length, respectively, both loci being separated by only 4.1–4.7 kb. However, in medaka (*Oryzias latipes*) only the *aqp1a *loci could be found in available genome sequence databases and it was 3.1 kb in length. In all teleosts analyzed, *aqp1a *and *aqp1b *were organized into 4 exons of 354–366 bp, 162–165 bp, 81 bp, and 177–204 bp in length, for exon 1, exon 2, exon 3, and exon 4, respectively. Comparison of the nucleotide sequence of exon 1, exon 2 and exon 3 between *aqp1a *and *aqp1b *within the same species showed 64–74% identity, while the sequence of exon 4, which encodes the C-terminal amino acid sequence, was only 57–58% identical.

**Figure 3:**
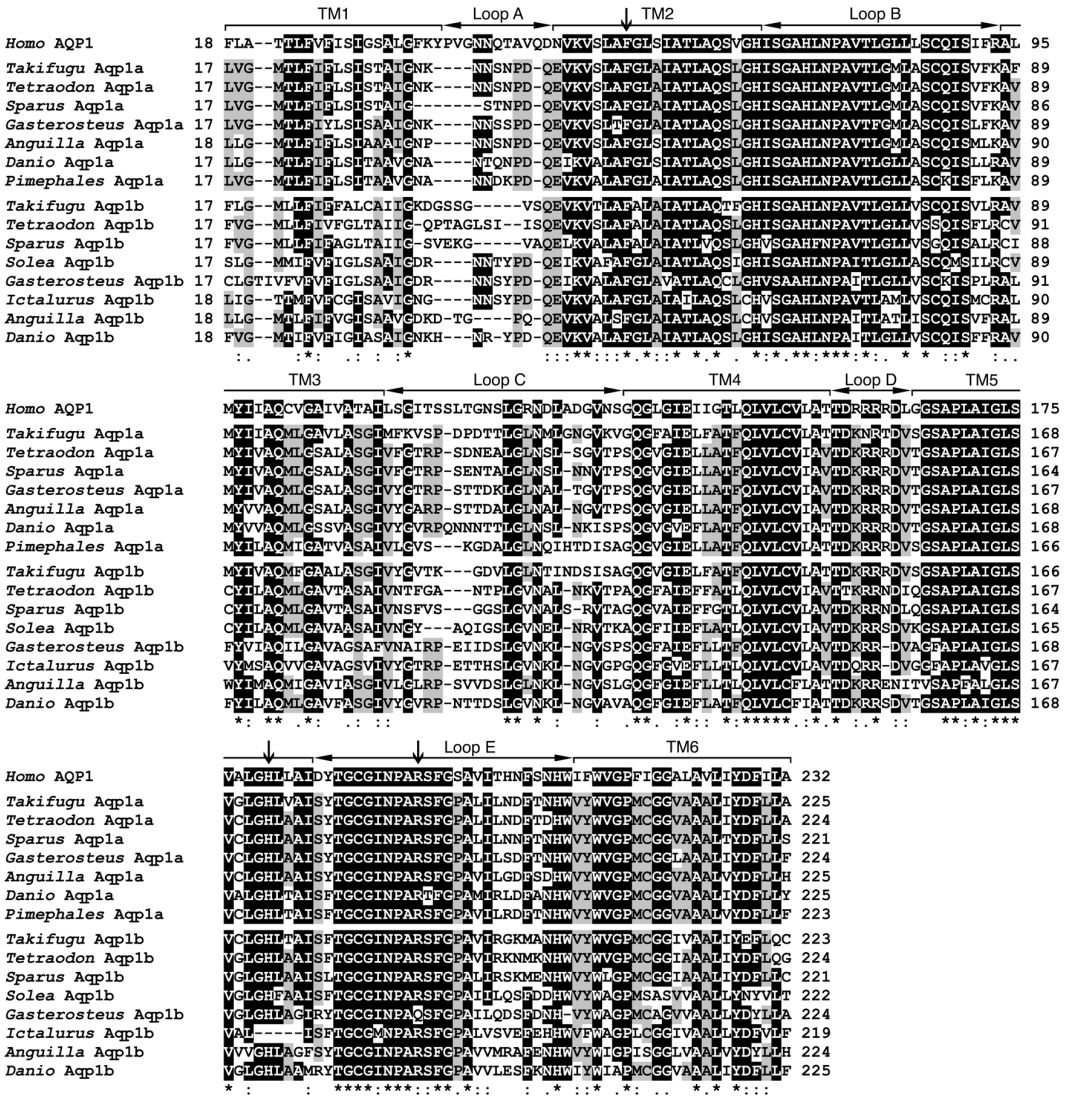
**Amino acid sequence alignment of human AQP1 and teleost Aqp1a and Aqp1b**. The six transmembrane (TM) domains and connecting loops A-B of human AQP1 are indicated by brackets and horizontal arrows, respectively. The vertical arrows above human AQP1 show the conserved residues Phe^56^, His^180 ^and Arg^195 ^(human AQP1 numbering) in water-selective AQPs. Identical residues between human AQP1 and teleost AQP1-like sequences are indicated with an asterisk, whereas conserved amino acid substitutions and substitutions with similar amino acids are indicated by a double or single dot, respectively. Residues conserved in human and most teleost sequences are shaded in black, and residues different from human but conserved between most of the teleost Aqp1a and Aqp1b sequences are shaded in grey.

The deduced Aqp1a amino acid sequence was slightly more similar to human AQP1 (60–61% identity) than that of Aqp1b (51–56% identity). Comparison of the primary structure of AQP1-like polypeptides between human and teleosts (Figure 3) indicated that Aqp1a and Aqp1b sequences have the six potential transmembrane domains and the two NPA motifs, as well as the residues of the pore-forming region (Phe^56^, His^180 ^and Arg^195^; human AQP1 numbering) that are conserved in water-selective AQPs [17]. In addition, in both Aqp1a and Aqp1b amino acid sequences, the Cys residue were before the second NPA motif (Cys^178 ^for sea bream Aqp1a and Aqp1b), which is the site in mammalian AQP1 potentially responsible for the inhibition of water permeability by mercurial compounds [18]. However, teleost Aqp1a and Aqp1b share only 61–64% identity, the lowest identity being at the level of the C-terminus (8–27%). The amino acid sequence of Aqp1a, including the C-terminus, among teleost species was relatively similar (69–95% identity), while that of Aqp1b appeared to be more divergent (51–76% identity among species) especially at the C-terminus (11–50% identity).

### Functional Aqp1b is predominantly expressed in the ovary of marine and catadromous teleosts

**Figure 4:**
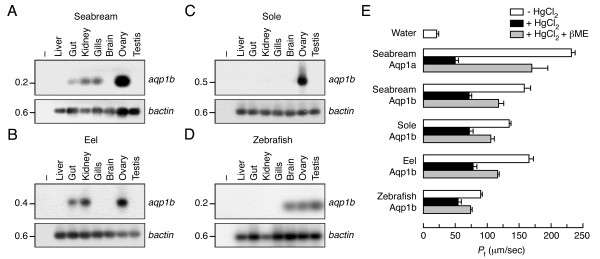
**Gene expression pattern and functional characterization of teleost Aqp1b**. (A-D) Representative RT-PCR analysis of *aqp1b *(upper panels) and *bactin *(lower panels) transcripts in sea bream (A), European eel (B), Senegalese sole (C) and zebrafish (D) tissues. PCR products were detected by Southern blot. Minus indicates absence of RT during cDNA synthesis. The size (kb) of PCR products and molecular markers are indicated on the left and right, respectively. (E) *P*_f _and Hg^2+ ^inhibition of *X. laevis *oocytes expressing teleost Aqp1a or Aqp1b. Oocytes were injected with cRNAs encoding sea bream Aqp1a or Aqp1b (1 ng), eel Aqp1b (10 ng), Senegalese sole Aqp1b (10 ng) or zebrafish Aqp1b (10 ng), or with 50 nl of water (control). The *P*_f _was assayed in the presence or absence of 0.7 mM HgCl_2_. Some oocytes treated with HgCl_2 _were incubated with 5 mM β-mercaptoethanol (βME) for 15 min before swelling measurements. Values represent the mean ± SEM (*n *= 6–10 oocytes) from a representative experiment.

Previous studies have reported that sea bream *aqp1b *mRNA is predominantly expressed in the ovary, whereas sea bream and eel *aqp1a *mRNA is ubiquitously expressed [10,19]. We investigated the expression pattern of *aqp1b *in teleosts belonging to different taxonomic groups that also have different reproductive strategies, such as Senegalese sole (Pleuronectiformes; marine), European eel (Anguilliformes; catadromous, i.e., live in freshwater and migrate to the sea to breed) and zebrafish (Cypriniformes; strict freshwater). The RT-PCR analysis confirmed that in pelagophil species, such as sea bream, sole and eel, *aqp1b *transcripts were highly abundant in mature (hydrated) ovaries, although it was also expressed in kidney, intestine and gills (Figure 4A–C). In contrast, in zebrafish *aqp1b *transcripts were detected at lower and similar levels in brain, ovary and testis (Figure 4D), thus showing a completely different expression pattern than in the other species. In zebrafish and sole, *aqp1a *mRNA was detected in all adult tissues examined, similarly to that reported in sea bream and eel (data not shown).

The highly conserved amino acid sequence of loops B and E of teleost Aqp1a and Aqp1b with respect to those of human AQP1 suggest that fish Aqp1b paralogs might encode functional water channels. To test this, *X. laevis *oocytes injected with cRNAs encoding sea bream Aqp1a or Aqp1b, sole Aqp1b, eel Aqp1b or zebrafish Aqp1b were compared with oocytes injected with 50 nl of water (Figure 4E). Coefficients of water osmotic permeability (*P*_f_) were determined from rates of oocyte swelling after transfer to hypoosmotic MBS. Water-injected oocytes exhibited low water permeability, whereas the *P*_f _of sea bream Aqp1a oocytes was increased by 10-fold, sea bream Aqp1b and eel Aqp1b oocytes by 8-fold, sole Aqp1b oocytes by 6-fold, and zebrafish Aqp1b oocytes by 4-fold. The presence of 0.7 mM HgCl_2 _reduced the *P*_f _of both Aqp1a- and Aqp1b-injected oocytes (82.6 ± 2.1% and 50.2 ± 3.1%, respectively). The inhibition was partially recovered (52.2 ± 8.3% and 27.5 ± 10.3%, for Aqp1a and Aqp1b, respectively) by incubation of oocytes with 5 mM β-mercaptoethanol.

### Sea bream Aqp1a and Aqp1b are differentially translocated into the oocyte plasma membrane

**Figure 5:**
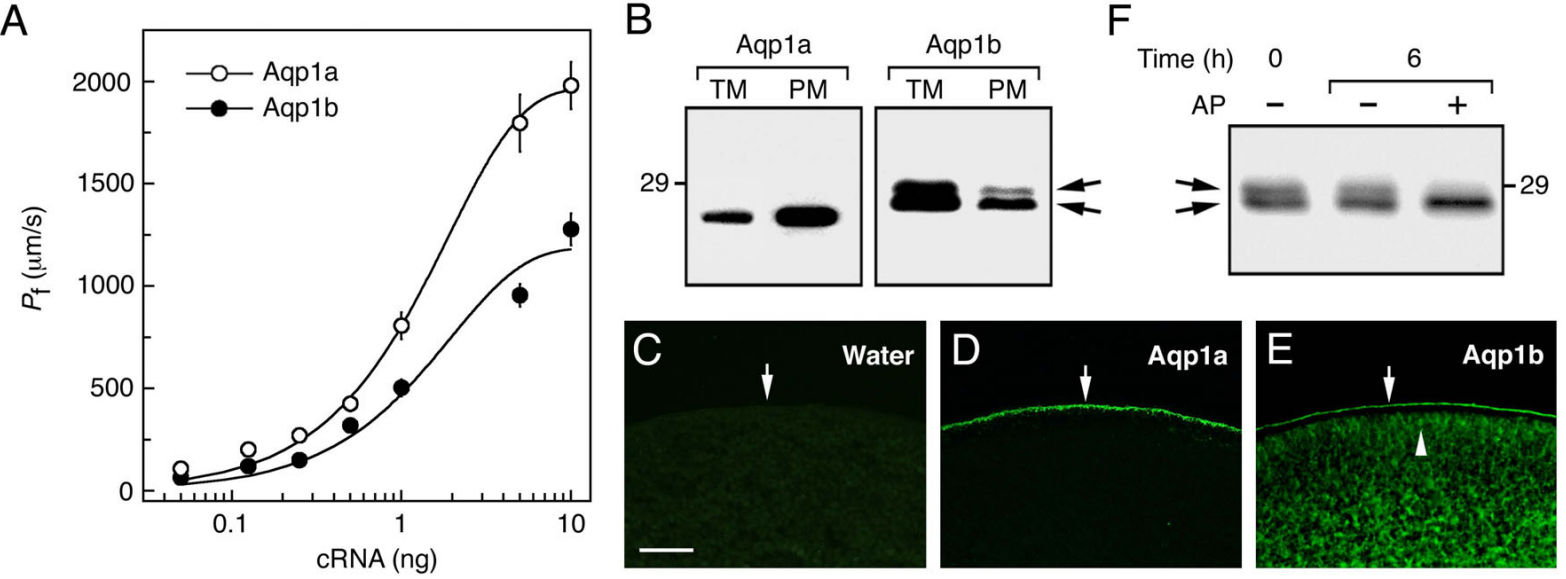
**Differential localization of sea bream Aqp1a and Aqp1b expressed in *X. laevis *oocytes**. (A) *P*_f _of oocytes expressing increasing amounts of Aqp1a or Aqp1b cRNA. Values represent the mean ± SEM (*n *= 6–10 oocytes) from 3 independent experiments. (B) Immunoblots of total and plasma membrane equivalents (TM and PM, respectively) of oocytes expressing 1 ng of Aqp1a or Aqp1b. The arrows indicate two very close Aqp1b reactive bands. (C-E) Immunofluoresence microscopy of water-injected and Aqp1a- or Aqp1b-expressing oocytes. Arrows show localization of the protein at the plasma membrane. The arrowhead indicates Aqp1b in the cytoplasm below the plasma membrane. Bar, 50 μm. (F) Immunoblot of total membrane fraction of Aqp1b-expressing oocytes incubated with or without alkaline phosphatase (AP) for 6 h. In B and F, the apparent molecular mass of a 29-kDa marker is indicated on the left and right, respectively.

Previous functional analyses indicated that oocytes expressing sea bream Aqp1a were more permeable than those expressing Aqp1b. To investigate whether both AQPs were differentially expressed or translocated in the oocyte cytoplasm, a series of dose-response experiments, injecting increasing amounts of Aqp1a or Aqp1b cRNA followed by Western blot and immunocytochemical analyses, were carried out (Figure 5). At all doses tested (from 0.05 to 10 ng cRNA), the *P*_f _of oocytes expressing Aqp1b was lower than that of oocytes expressing Aqp1a (Figure 5A), while protein expression levels resulting from both cRNAs were similar (Figure 5B, lane TM). Immunocytochemistry on sections of these oocytes revealed that Aqp1a was present in the plasma membrane (Figure 5D), whereas Aqp1b was also detected just below the plasma membrane, possibly in vesicles (Figure 5E). This staining was specific for Aqp1b since control oocytes, not injected or injected with water, were unstained (Figure 5C). Western blots of protein extracts from total and plasma membranes of oocytes showed there was a higher proportion of Aqp1a in the plasma membrane when compared with Aqp1b (Figure 5B), confirming that Aqp1b was retained in the cytoplasm. However, immunoblots of total and plasma membrane of Aqp1b-expressing oocytes revealed the presence of two reactive bands of approximately 28 and 29 kDa after incubation with the Aqp1b antisera (Figure 5B, arrows). Both bands were visible in total and plasma membrane fractions, although the 29-kDa band was much weaker in the plasma membrane fraction. Alkaline phosphatase treatment of total membrane proteins before SDS-PAGE prevented the detection of the 29-kDa polypeptide, indicating that this band corresponded to a phophorylated form of Aqp1b (Figure 5F).

### Role of sea bream Aqp1b C-terminus for Aqp1b cell surface expression

**Figure 6:**
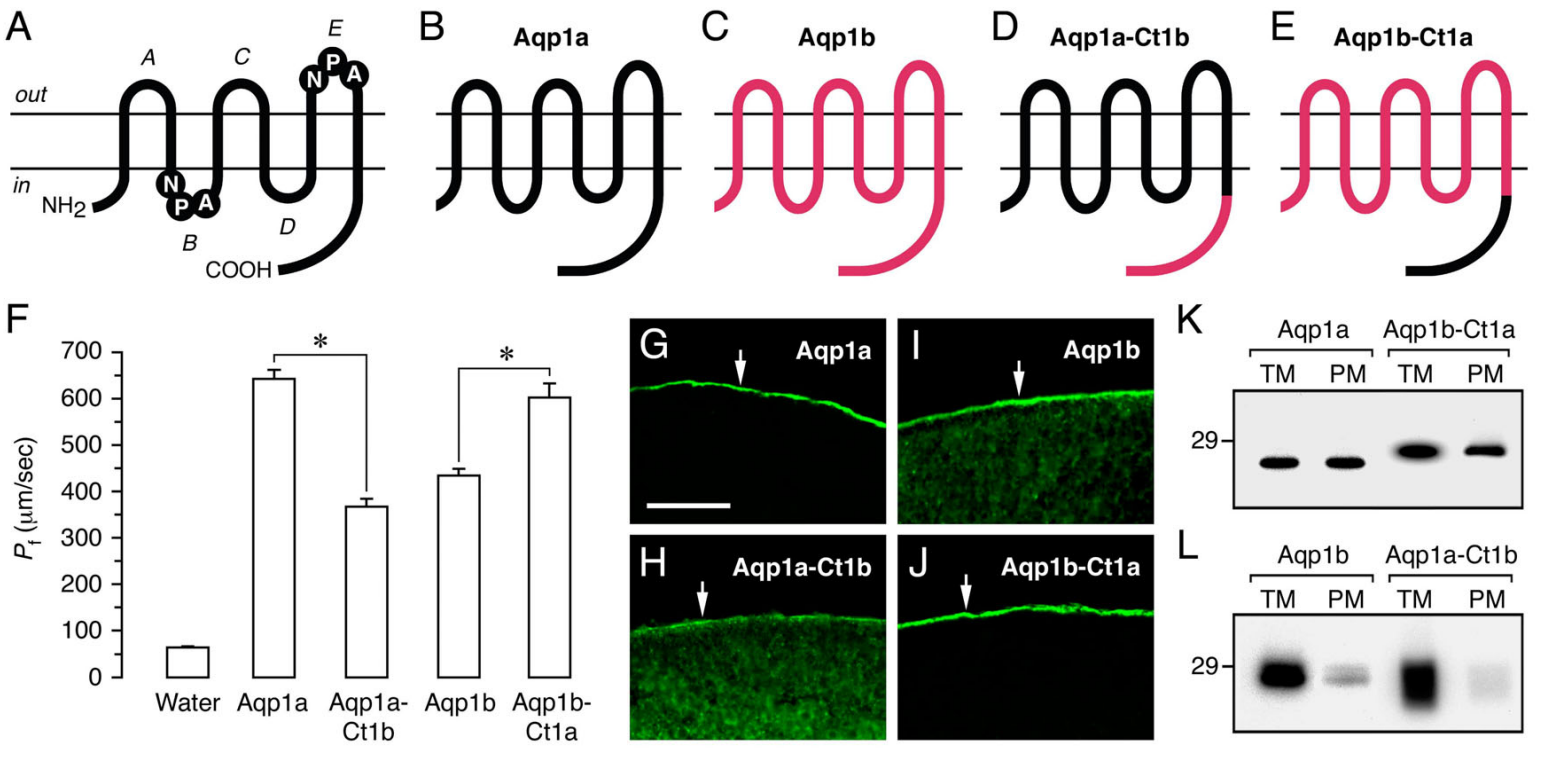
**Functional properties and subcellular localization of wild-type (WT) sea bream Aqp1a and Aqp1b, and of their chimeric proteins, in oocytes**. (A) Membrane topology of AQP family members showing the six transmembrane helices with five connecting loops (*A*-*E*), and two conserved Asn-Pro-Ala (NPA) motifs in loops *B *and *E*. (B-E) WT Aqp1a (B) and Aqp1b (C), Aqp1a chimera in which the C-terminus of Aqp1a was exchanged with that of Aqp1b (Aqp1a-Ct1b; D), and Aqp1b chimera in which the C-terminus of Aqp1b was exchanged with that of Aqp1a (Aqp1b-Ct1a; E). (F) *P*_f _of oocytes expressing 1 ng of cRNA encoding WT or chimeric proteins. Values represent the mean ± SEM (*n *= 5–8 oocytes) from a representative experiment. The asterisk denotes statistically significant differences (*p *< 0.01). (G-J) Immunofluorescence microscopy of oocytes localizing WT Aqp1a and Aqp1b-Ct1a exclusively at the plasma membrane, whereas WT Aqp1b and Aqp1a-Ct1b are also in the cytoplasm. Sections shown in G and J were probed with the anti-Aqp1a antisera, whereas the sections in H and I were probed with the anti-Aqp1b antisera. Bar, 50 μm. (K-L) Immunoblots of total and plasma membrane equivalents (TM and PM, respectively) of oocytes expressing the differents cRNAs. Blots were probed as indicate above. The apparent molecular mass of a 29-kDa marker is indicated on the left.

As indicated, the most divergent region between the amino acid sequence of sea bream Aqp1a and Aqp1b was the C-terminus. Since it is known that the cytoplasmic tail plays a role in the intracellular trafficking of some mammalian [20] and amphibian [21] AQPs, we studied whether this region was involved in the control of Aqp1b translocation into the oocyte plasma membrane. Expression vectors were made in which either the C-terminal tail of Aqp1a was exchanged with that of Aqp1b (Aqp1a-Ct1b), or the C-terminus of Aqp1b with that of Aqp1a (Aqp1b-Ct1a), in both cases starting at Pro^222^. The two chimeras, as well as wild-type Aqp1a and Aqp1b, were expressed in oocytes and the *P*_f _and protein expression at the oocyte plasma membrane monitored (Figure 6A–E). Expression of Aqp1a-Ct1b reduced swelling by 53.8 ± 5.2% when compared with oocytes expressing wild-type Aqp1a, whereas water permeability of oocytes expressing Aqp1b-Ct1a was increased by 37.1 ± 8.1% with respect to wild-type Aqp1b-expressing oocytes (Figure 6F). Oocyte permeability positively correlated with accumulation of intact and chimera AQP in the plasma membrane, with both Aqp1a and Aqp1b-Ct1a being exclusively localized at the membrane (Figure 6G and 6J), whereas Aqp1b and Aqp1a-Ct1b were also present in the cytoplasm (Figure 6H and 6I). Western blot of total and plasma membranes revealed the presence of a single 27-kDa band in Aqp1a and Aqp1b-Ct1a oocytes, while Aqp1b and Aqp1a-Ct1b oocytes had the 28- and 29-kDa bands or a smear band of 27 to 29 kDa, respectively (Figure 6K and 6L). These observations suggest that retention of Aqp1b in the oocyte cytoplasm may be associated to phosphorylation at the level of the C-terminus.

### In silico analysis of teleost Aqp1b C-terminal amino acid sequence

**Figure 7:**
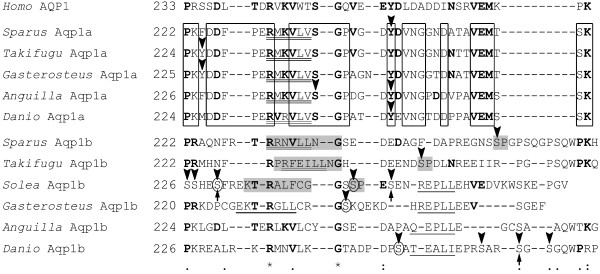
**Amino acid sequence alignment of the C termini of human AQP1, and Aqp1a and Aqp1b from representative teleosts**. At the bottom, identical residues are indicated by asterisks, whereas conserved amino acid substitutions and substitutions with similar amino acids are indicated by double or single dots, respectively. Residues of teleost Aqp1a and Aqp1b conserved in human AQP1 are in bold, and conserved residues in Aqp1a sequences are boxed. Double underlined residues indicate typical potential sorting and internalization sequences, whereas those single underlined indicate other sorting-like motifs. In each sequence, potential Ser, Thr and Tyr phosphorylation sites (score ≥ 0.9) are indicated by arrowheads. Consensus sites for potential kinases are indicated: grey, candidate Pro-directed kinase and preceding docking domain; arrows, casein kinase I (CK1); circles, CK2. Other candidate phosphorylation sites shown do not match any eukaryotic linear functional motif included in the ELM resource.

The C-terminal amino acid sequence of Aqp1a and Aqp1b from different teleosts was searched for potential regulatory sites (Figure 7). Unlike human AQP1, both Aqp1a and Aqp1b sequences showed typical sorting and internalization motifs ([D/E/R]XXXL [L/V/I]) in the cytoplasmic tail, common in transmembrane proteins [22]. These signals appeared to be highly conserved in fish Aqp1a (with the consensus sequence R [M/V] [K/R]VLV), whereas in Aqp1b they were more variable, although all of them included a di-Leu or Leu-Ile motif. Teleost Aqp1a and Aqp1b also had a variable number of Ser, Tyr and Thr residues with a high probability of being phosphorylated. Among these residues, Ser^254 ^in sea bream Aqp1b had a high phosphorylation score (0.98) and fulfilled the criteria for a Pro-directed kinase phosphorylation site ([S/T]P preceded by a docking domain [R/K]XXXXØXØ; Ø denotes hydrophobic residue; [23,24]). The same consensus site was found in the C-terminus of fugu (Ser^245^; score 0.97) and sole (Ser^230^; score 0.95) Aqp1b, but not in that of eel, stickleback or zebrafish Aqp1b, although these sequences, except that of eel, had one or more Ser residues with a high phosphorylation score (≥ 0.9). Some of these residues matched a casein kinase 1 (CK1) phosphorylation site (Ser^230 ^and Ser^260^, in sole and zebrafish Aqp1b, respectively), or a CK2 site (Ser^230^, Ser^237 ^and Ser^247^, in sole, stickleback and zebrafish, respectively). In addition, a Thr residue preceding the sorting signal appeared to be conserved only in teleost Aqp1b sequences (Thr^229^, Thr^235^, Thr^229 ^and Thr^230^, in sea bream, sole, stickleback and eel, respectively), except in fugu and zebrafish, where it was replaced by Phe^227 ^and Lys^233^, respectively. However, none of these Thr residues gave a relevant phosphorylation score.

### Involvement of specific residues in sea bream Aqp1b C-terminus in intracellular trafficking

**Figure 8:**
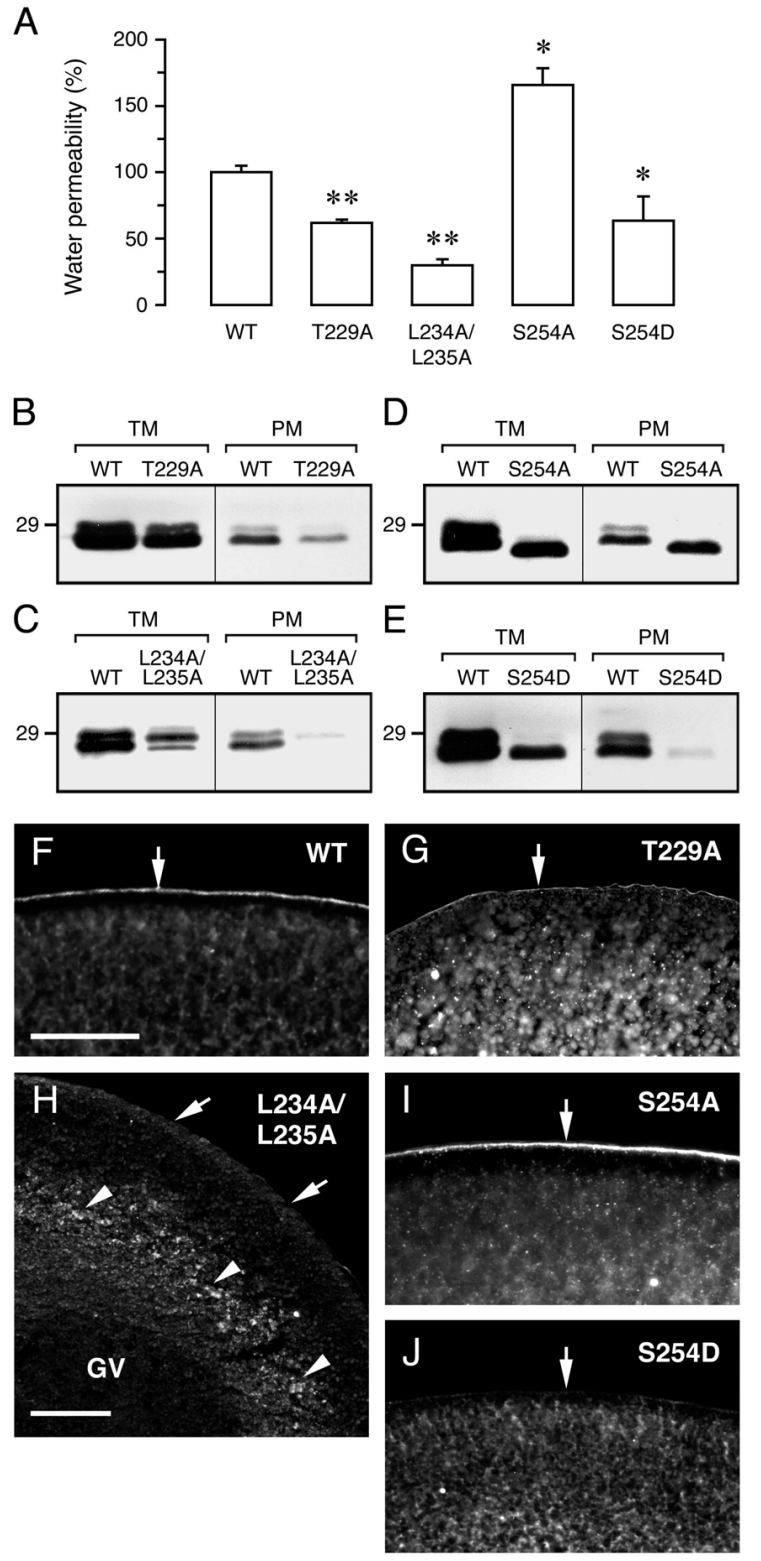
**Role of specific residues in the sea bream Aqp1b C-terminal tail for intracellular trafficking in oocytes**. (A) Water permeability of oocytes expressing wild-type (WT) or mutant Aqp1b. Oocytes were injected with cRNAs encoding WT Aqp1b (0.25 or 1 ng), Aqp1b-T229A (1 ng), Aqp1b-L234A/L235A (1 ng), Aqp1b-S254A (0.25 ng) or Aqp1b-S254D (0.25 ng). Permeability is expressed in % related to oocytes injected with WT Aqp1b. Values are the mean ± SEM of 3–5 experiments (n = 10–15 oocytes per treatment). The asterisks denote statistically significant differences (*, *p *< 0.05; **, *p *< 0.01). (B-E) Immunoblots of total and plasma membrane equivalents (TM and PM, respectively) of oocytes expressing WT or mutant Aqp1b. The apparent molecular mass of a 29-kDa marker is indicated on the left. (F-J) Localization of Aqp1b mutants in oocytes. The plasma membrane is indicated by arrows, and retention of Aqp1b-L234A/L235A proteins possibly in the ER is indicated by arrowheads (H). Bars, 100 μm.

To investigate the potential role of the semi-conserved C-terminal Ser and Thr residues in Aqp1b intracellular trafficking, sea bream Aqp1b Ser^254 ^and Thr^229 ^were independently mutated into Ala or Asp to mimic non-phosphorylated and phosphorylated states, respectively (Table 1). The Leu^234^Leu^235 ^motif in this sequence was also mutated into an Ala pair. Wild-type and mutated proteins were then expressed in oocytes to determine their permeability and subcellullar localization as indicated above (Figure 8). Swelling assays showed that Aqp1b-T229A and Aqp1b-L234A/L235A mutants reduced water permeability by 38.1 ± 2.4% and 70.1 ± 4.4%, respectively, with respect to oocytes expressing wild-type Aqp1b (Figure 8A). For Aqp1b-T229A mutant, reduced permeability was apparently caused by retention of the protein in cytoplasmic vesicles, although it did not affect the phosphorylation state of Aqp1b (Figure 8B and 8G). In contrast, as the mutant was predominantly localized surrounding the oocyte nucleus (i.e., germinal vesicle), the strong inhibition of water permeability showed by Aqp1b-L234A/L235A was possibly caused by the accumulation of the protein in intracellular compartments, which could enhance protein-lysosomal targeting and degradation (Figure 8C and 8H). The Aqp1b-T229D mutant was less expressed than wild-type Aqp1b (data not shown), causing low accumulation of the protein in the plasma membrane, thereby reducing oocyte water permeation (by 63.4 ± 4.0% inhibition). However, water permeability was higher in oocytes expressing the Aqp1b-S254A mutant than those expressing wild-type Aqp1b (65.6 ± 12.8% increase), associated with the absence of a phosphorylated form of the protein and increased expression in the plasma membrane (Figure 8A, D and 8I). Conversely, Aqp1b-S254D induced the retention of the protein in cytoplasmic vesicles as with Aqp1b-T229A, thus inhibiting protein translocation into the plasma membrane and reducing water permeability (36.7 ± 18.5% reduction) (Figure 8A, E and 8J). Other Ser residues present in the C-terminal region of sea bream Aqp1b, Ser^238^, Ser^253^, Ser^258 ^and Ser^262^, were also substituted by Ala but these mutants had no effect on water permeability with respect to wild-type Aqp1b (see Additional file 2). These data suggest that dephosphorylation of Ser^254 ^may enhance Aqp1b shuttling into the plasma membrane, and that Thr^229 ^may also regulate this process through a phosphorylation-independent mechanism.

**Table 1:** C-Terminal amino acid sequences of sea bream wild-type (WT) and mutated Aqp1b

Construct	Aqp1b C-terminus sequence
	222 267
	| |
Aqp1b-WT	PRAQNFR**T**RRNV**LL**NG**S**EDEDAGFDAPREGN**SS**PGP**S**QGP**S**QWPKH
T229A	PRAQNFR**A**RRNVLLNGSEDEDAGFDAPREGNSSPGPSQGPSQWPKH
T229D	PRAQNFR**D**RRNVLLNGSEDEDAGFDAPREGNSSPGPSQGPSQWPKH
L234A/L235A	PRAQNFRTRRNV**AA**NGSEDEDAGFDAPREGNSSPGPSQGPSQWPKH
S238A	PRAQNFRTRRNVLLNG**A**EDEDAGFDAPREGNSSPGPSQGPSQWPKH
S253A	PRAQNFRTRRNVLLNGSEDEDAGFDAPREGN**A**SPGPSQGPSQWPKH
S254A	PRAQNFRTRRNVLLNGSEDEDAGFDAPREGNS**A**PGPSQGPSQWPKH
S254D	PRAQNFRTRRNVLLNGSEDEDAGFDAPREGNS**D**PGPSQGPSQWPKH
S258A	PRAQNFRTRRNVLLNGSEDEDAGFDAPREGNSSPGP**A**QGPSQWPKH
S262A	PRAQNFRTRRNVLLNGSEDEDAGFDAPREGNSSPGPSQGP**A**QWPKH

Amino acid sequence of C termini of WT and Aqp1b mutants from Pro^222 ^to terminal His^267^. Amino acid substitutions are shown in black boxes.

## Discussion

We present here strong evidence confirming the presence of two AQP isoforms in vertebrates that are structurally and functionally related to mammalian AQP1. These isoforms, Aqp1a and Aqp1b, seem to coexist exclusively in teleost fish since Aqp1b was not found in mammals, amphibians or birds. The main difference between Aqp1a and Aqp1b is in the C-terminal tail, which contains specific residues for regulation of intracellular trafficking in Aqp1b.

Previous sequence analyses of MIPs suggest that substrate selective modes (AQPs and aquaglyceroporins) were acquired early in the history of the family by gene duplication and functional shift, with the highest level of diversification occurring in vertebrates and higher plants [25]. Analysis of Aqp1a and Aqp1b distribution by searching currently available genome sequence information and by cDNA cloning suggest that both isoforms are present exclusively in the teleost genome. Phylogenetic reconstruction of vertebrate AQP1-like proteins indicates that Aqp1a and Aqp1b share a common origin and are likely to have evolved from duplication of a common ancestor. Because both isoforms are present in fish species belonging to distant taxonomic groups, from basal (e.g., Anguilliformes) to more modern (e.g., Gasterosteiformes, Perciformes, Pleuronectiformes and Tetraodontiformes) groups [26,27], this duplication must be ancient and is likely to have affected most teleosts. As suggested for many duplicated genes in teleosts, the origin of Aqp1b might be the whole-genome duplication (WGD) event that occurred specifically in the ray-finned (Actinopterygian) lineage after splitting from the tetrapod lineage about 350 million years ago [28]. However, as it has not been possible to identify Aqp1b in most basal actinopterygians (e.g., paddlefish and sturgeon) with the genomic information available, it is not known whether the AQP1 duplication also affected these groups. In all teleosts examined, the *aqp1a *and *aqp1b *loci were found to be closely linked, indicating that Aqp1b possibly arose by a gene duplication event at a local level rather than at the chromosome or genome level. Local gene duplication has also been proposed, for instance, to explain the repertoire of teleost opsins [29,30] and the generation of the *Xiphophorus Xmrk *oncogene [31].

The radiation of teleosts in the ocean most likely required the evolution of new osmoregulatory mechanisms in eggs and early embryos to alleviate the passive water loss imposed by the hyper-osmotic environment [7]. In this scenario, it is plausible to hypothesize that duplication of *aqp1 *genes in teleosts allowed for one duplicate to encode a product with a new function through innovating mutations in regulatory and/or structural sequences ('neofunctionalization'). This seems to be the case for sea bream Aqp1b which plays a specialized physiological role in the oocyte mediating water uptake during meiotic maturation [10,11]. In other marine and catadromous teleosts, such as sole and eel, respectively, that like sea bream also spawn highly hydrated eggs, we show here that *aqp1b *also encodes a functional water channel whose RNA is predominantly accumulated in the ovary, suggesting a similar role of Aqp1b during oocyte hydration. In other pelagophil teleosts [32,33], as well as in some species of catfish, in which oocyte hydration may also occur despite having a freshwater life cycle (e.g., [34]), Aqp1b-encoding ESTs have also been found in the ovary. In contrast, in zebrafish, a freshwater species where almost no oocyte hydration is observed [35], we found a completely different *aqp1b *expression pattern, together with a higher mutation rate in the amino acid sequence of the encoded protein. Based on these data, we argue that, in marine teleosts producing highly hydrated eggs, Aqp1b possibly represents a neofunctionalized water channel adapted to oocytes to facilitate water transport. Finn and Kristoffersen [7] recently proposed that neofunctionalization of duplicated Vtg genes, which allowed one paralog to be proteolyzed into FAAs driving hydration of the maturing oocytes, was a key event in the evolution and success of the teleosts in the oceanic environment. The duplication and neofunctionalization of *aqp1b *may have occurred in parallel to this mechanism to facilitate oocyte water uptake in marine teleosts.

The Aqp1b isoform found in freshwater teleosts that spawn non-hydrated eggs, such as zebrafish, might be inactivated by mutations, or be eventually lost in the genome. This hypothesis is supported by the absence of *aqp1b *in advanced freshwater species, such as medaka, while the synteny between the *aqp1a *chromosome loci and downstream genes (e.g., *thoc1*) is conserved. However, in other extant freshwater teleosts that arose later in evolution, such as *Tetraodon*, *aqp1b *is retained in the genome. The retention of *aqp1b *in freshwater pufferfishes is intriguing, given that there appears to have been a massive elimination of DNA after WGD in most modern teleost genomes, resulting in the retention of only a subset of the duplicates [36,37]. It is possible to speculate, however, that the recent evolution of Tetraodontiformes did not last long enough to allow specific divergence of the genome and hence of *aqp1b*. The relatively high amino acid sequence identity (77%) between fugu, a marine pufferfish which produces hydrated eggs, and *Tetraodon *Aqp1b supports this conjecture. In any event, additional studies aiming at the characterization of Aqp1b in more freshwater fish species, as well as the determination of sites of gene expression and protein accumulation, are required to better understand the driving force behind *aqp1b *isoform evolution.

In addition to the ovary, *aqp1b *mRNA has been detected in the posterior intestine, kidney, gills and esophagus of marine fish (this work, and [10,14,38]). The intestine of marine teleosts has an important osmoregulatory role, as hypo-osmoregulating fish have long been known to drink seawater to replace water lost by diffusion to their environment (see [39] for review). Accordingly, Aqp1a and Aqp1b proteins have been reported to be localized in the intestinal epithelia of teleosts [12-15]. However, in the sea bream gastrointestinal tract Aqp1a and Aqp1b have a different distribution pattern. Whereas Aqp1a is localized in the apical and basolateral membrane of enterocytes in duodenum and hindgut, Aqp1b is exclusively localized in the apical microvilli of rectal epithelial cells [12]. Moreover, freshwater acclimation reduces the synthesis of Aqp1a in all intestinal segments, and of Aqp1b in rectum. Conversely, seawater acclimation of eels increases Aqp1a expression and protein synthesis in the intestine [13,19]. Therefore, although the specific physiological functions of Aqp1a and Aqp1b in the teleost gastrointestinal tract remain unknown, these data may point to an additional role of Aqp1b in water movement across the intestinal epithelia.

The primary structure of AQP1-like proteins corresponding to the TM2 and TM5 domains and connecting loops B and E, which are involved in the formation of the water-selective pore, is highly conserved between teleost and mammals. However, Aqp1a and Aqp1b show different permeability efficiencies when expressed in *X. laevis *oocytes, and teleost Aqp1b isoforms also show a marked structural divergence at the C-terminal cytoplasmic tail with respect to Aqp1a and mammalian AQP1. Functional experiments, using artificial expression in oocytes of sea bream wild-type Aqp1a and Aqp1b, and chimeric proteins in which the C-terminus of Aqp1a was totally exchanged for that of Aqp1b, or the reverse, revealed that the Aqp1a tail drives constitutive targeting to the plasma membrane, unlike that of Aqp1b which produces partial retention of the expressed proteins in intracelluar vesicles. These data strongly suggest that Aqp1b independently acquired specific regulatory domains in the C-terminal region for the control of Aqp1b intracellular trafficking.

To investigate the nature of putative regulatory sites in the Aqp1b C-terminus, we analyzed its amino acid sequence in different teleosts. Based on this analysis, selected residues of sea bream Aqp1b were mutated into Ala or Asp and the resulting proteins were expressed in oocytes to determine their intracellular localization and permeability properties. In the Aqp1b C-terminus, we found typical sorting and internalisation signals that are common in many mammalian transmembrane proteins for targeting from the trans-Golgi network to the lysosomal-endosomal compartment [22]. These motifs, however, were also detected in the Aqp1a C-terminal tail, although their sequence appeared to be different between the Aqp1a and Aqp1b isoforms. Thus, in all six teleost Aqp1b sequences analyzed, but not in Aqp1a, a di-Leu or Leu-Ile signals appear to be conserved. Mutation of sea bream Aqp1b Leu^234^Leu^235 ^motif into Ala^234^Ala^235 ^produced the retention of the protein in intracellular compartments and apparently increased its degradation, thereby reducing water permeability of these oocytes. These results are similar to those observed with the mammalian AQP2 mutant which has an altered and extended C-terminal tail, retained in late endosomes/lysosomes triggering degradation [40]. Similarly, mutation of the C-terminal Leu^345^Leu^346 ^motif in the human vasopressin V3 receptor produces mis-folding of the protein and abolishes receptor export [41]. It is possible therefore, that the di-Leu motif in sea bream Aqp1b also plays a role in conformation, ensuring correct routing to the plasma membrane. However, di-Leu motifs are also involved in basolateral membrane targeting and microvilli anchoring of mammalian cell adhesion proteins, ion channels and receptors [42-45], including AQP4 [46], in polarized epithelial cells. Further studies are needed to establish whether the di-Leu motif has an additional function in the control of Aqp1b expression on the cell surface.

Most notably, functional analyses revealed that two residues in the sea bream Aqp1b C-terminal sequence, Thr^229 ^and Ser^254^, were responsible for sea bream Aqp1b translocation from intracellular vesicles to the oocyte plasma membrane. The probability of the Thr^229 ^residue being phosphorylated was low, and accordingly the Aqp1b-T229A mutant did not affect the phosphorylation state of Aqp1b, although it did inhibit Aqp1b cell surface expression and oocyte water permeability. Since Thr^229 ^did not match any kinase phosphorylation consensus site other than protein kinase C (which apparently is not relevant here), the specific function of this residue is unknown and awaits further experimentation. Nevertheless, it was observed that the Aqp1b-S254A mutant prevented phosphorylation and increased Aqp1b translocation into the plasma membrane and subsequent water permeability, whereas the Aqp1b-S254D mutant, which mimicked the constitutively phosphorylated state of Aqp1b, was predominantly located in intracellular vesicles. These results suggest that dephosphorylation of Ser^254 ^triggers Aqp1b shuttling to the cell surface, while its phosphorylation may retain the protein in intracellular vesicles. This mechanism is thus apparently the opposite to that described so far for mammalian and amphibian AQPs (i.e., AQP2 and AQP-h2), where channel insertion in the plasma membrane of collecting duct cells or granular cells of the anuran urinary bladder is triggered by protein kinase A-mediated phosphorylation of specific Ser residues in the C-terminal tail [21,47]. Interestingly, the Ser^254 ^in sea bream Aqp1b, a consensus site for a Pro-directed kinase, seems to be conserved in modern marine teleosts which produce hydrated eggs (Ser^244 ^in fugu Aqp1b and Ser^244 ^in sole Aqp1b). Pro-directed kinases are a large family of mitogen-activated protein kinases (MAPK) and cyclin-dependent kinase-like kinases, some of which (e.g., p38 MAPK) are involved in transduction pathways leading to the activation of the maturation promoting factor (MPF) during oocyte meiotic maturation [48-50]. In sea bream, Aqp1b translocation into the oocyte plasma membrane is a tightly regulated process thought to occur transiently downstream of MPF activation during meiotic maturation, just before complete hydrolysis of yolk proteins and maximum K^+ ^accumulation is reached in the oocyte [11]. Therefore, it will be of interest to investigate the potential role of cell-cycle related kinases, or other kinases activated during oocyte maturation, in Ser^254 ^phosphorylation and regulation of Aqp1b trafficking.

## Conclusion

We provide phylogenetic and functional evidence for the teleost lineage-specific duplication of AQP1 channels and further divergence at the C-terminal tail. The generation and neofunctionalization of the Aqp1b isoform in oocytes of marine teleosts most likely contributed with the production of highly hydrated eggs to ensure survival in seawater. The neofunctionalization of Aqp1b has also been accompanied by the acquisition of regulatory domains in the cytoplasmic C-terminal tail for the specific control of Aqp1b intracellular trafficking, which are currently being investigated. The elucidation of the biological functions of Aqp1a and Aqp1b in teleosts will contribute to our understanding of the evolution of phenotypic complexity, diversity and innovation in vertebrates.

## Methods

### Animals

Adult zebrafish, gilthead sea bream, Senegalese sole, and European eel were purchased from local pet stores or fish farms and maintained as described [11,51-53]. Naturally spawning fish, or hormone-stimulated in the case of eel (see [53] for details), were sedated by immersion for approximately 15 min in 100 ppm phenoxyethanol, sacrificed by decapitation, and samples of mature ovary and other tissues immediately dissected and frozen at -80°C. Procedures relating to the care and use of animals were approved by the Ethics Committee from IRTA in accordance with the Guiding Principles for the Care and Use of Laboratory Animals.

### Cloning and sequencing of teleost AQP1-like cDNAs

Partial cDNAs encoding European eel Aqp1b and Senegalese sole Aqp1a were isolated by reverse transcriptase-polymerase chain reaction (RT-PCR) employing degenerate oligonucleotide primers (see Additional file 3). Total RNA was extracted from kidney, intestine and hydrated ovaries using the RNeasy Maxikit (Qiagen), followed by polyA RNA purification with the Oligotex mRNA Minikit (Qiagen). PolyA RNA (500 ng) was reverse transcribed using 0.5 μg oligo (dT)_17_, 1 mM dNTPs, 40 IU RNAse inhibitor (Roche), and 10 IU MMLuV-RT enzyme (Roche), for 1.5 h at 42°C. The PCR was carried out with 0.5 μl of the RT reaction in a volume of 50 μl containing 1 × PCR buffer plus Mg^2+^, 0.2 mM dNTPs, 0.2 μM of each forward and reverse oligonucleotide primers, and 1 IU of Taq polymerase (Roche). Reactions were amplified using one cycle of 95°C, 5 min; then 40 cycles of 95°C, 30 sec; 54°C, 30 sec; 72°C, 1 min; and a final 7-min elongation at 72°C. The products were cloned into the pGEM-T Easy Vector (Promega) and sequenced by BigDye Terminator version 3.1 cycle sequencing on ABI PRISM 377 DNA analyzer (Applied Biosystems). Full-length eel Aqp1b cDNA was isolated by rapid amplification of cDNA ends (RACE; Gibco) followed by a final amplification with a high-fidelity polymerase (Pwo; Roche). Full-length zebrafish Aqp1b cDNA was amplified from total RNA extracted from adult brain using specific forward and reverse primers based on a predicted cDNA (see Additional file 3). The nucleotide sequence of Senegalese sole Aqp1a, European eel Aqp1b, and zebrafish Aqp1b have been deposited in the GenBank database under accession numbers DQ889223, EF011738, and EU327345, respectively.

### Genomic organization of teleost *aqp1a *and *aqp1b *genes

Genomic sequences covering the complete sea bream *aqp1a *and *aqp1b *loci, as well as the flanking cassette, were amplified by PCR on liver-extracted genomic DNA using the Expand Long Template PCR system 3 (Roche) and gene specific primers (see Additional file 3). Products were cloned into the pGEM-T Easy Vector and sequenced as described. The nucleotide sequence of sea bream *aqp1a *and *aqp1b *loci have been deposited in the GenBank database under accession numbers EF011739 and EF011740, respectively. Zebrafish, medaka, fugu, pufferfish, and three-spined stickleback *aqp1a *and *aqp1b *genomic sequences were retrieved from ENSEMBL [54]. The exon-intron structure was determined from cloned cDNAs and available ESTs.

### Phylogenetic and sequence analyses

Vertebrate and teleost MIP sequences were retrieved from the NCBI database [55] and ENSEMBL and analyzed at the amino acid level. Amino acid sequence alignments were performed using the ClustalW multiple sequence alignment program [56] employing the sequence from the first NPA motif to the start of the C-terminus (when sequence data was available), and were manually optimized using the Bioedit software [57]. The alignment is shown in the Additional file 1. The phylogenetic tree and branch support values were estimated by using the NJ, ML and BI methodologies of phylogenetic reconstruction. The NJ analysis [58] of the amino acid alignment was based on mean character distances using Mega3 software [59]; bootstrap support values were obtained with 1,000 repetitions. For ML and BI analyses, a Bayesian consensus tree for the sequence data set was built and used to estimate the best-fit evolutionary model by using ProtTest v1.4 [60]. Then, ML (including bootstrapping) analysis was performed with PhyML [61]. To confirm the ML tree, a BI (including posterior probabilities) of phylogeny was conducted by using MrBAYES v3.1 [62] with the ProtTest best-fit model of amino acid substitution (CpRev) provided in the package. Four independent runs, each with four simultaneous Markov Chain Monte Carlo chains, were performed for 1,000,000 generations sampled every 100 generations. Potential Ser, Thr and Tyr phosphorylation sites in amino acid sequences were predicted using NetPhos 2.0 [63]. Candidate functional sites were identified using the Eukaryotic Linear Motif (ELM) server [64].

### Gene expression analysis

The abundance of *aqp1b *transcripts in sea bream, eel, Senegalese sole and zebrafish adult tissues was assessed by conventional RT-PCR followed by Southern blot. Total RNA from liver, intestine, kidney, gills, brain, ovary and testis was extracted, treated with DNase, and first-strand cDNA synthesized as described above. The PCR was carried out as above on 1 μl of the RT reaction using species-specific *aqp1b *forward and reverse oligonucleotide primers located in exon 3 and 4, respectively (see Additional file 3). For zebrafish, 500 ng of DNA template was also amplified using the corresponding oligos (not shown). In all experiments, β-actin was used as a reference gene; forward and reverse oligonucleotide primers designed in highly conserved regions of zebrafish β-actin1 (*bactin1*) (Additional file 3) were employed for all species. PCR reactions were performed with an initial cycle of 95°C, 5 min; then variable number of cycles and temperatures for amplification, depending on the species, to generate half-maximal amounts of PCR products (not shown); and a final 7-min elongation at 72°C. For sea bream aqp1b, the cycles were 28 of 95°C, 30 sec; 62°C, 30 sec; 72°C, 30 sec; for eel and Senegal sole *aqp1b *the cycles were 37 and 36, respectively, of 95°C, 1 min; 60°C, 1 min; 72°C, 1 min; and for zebrafish *aqp1b *the cycles were 35 of 95°C, 1 min; 65°C, 1 min; 72°C, 1 min. For *bactin1 *the cycles were 26 for sea bream and eel, 24 for sole, and 22 for zebrafish, of 95°C, 30 sec; 52°C, 30 sec; 72°C, 45 sec. The PCR products were electrophoresed on 1% agarose gels, and the DNA blotted to nylon membranes (Amersham). Membranes were hybridized with species-specific digoxigenin-labelled *aqp1b *probes using the DIG DNA Labelling Mix (Roche).

### Expression constructs

Sea bream Aqp1a and Aqp1b, and zebrafish, eel and Senegalese sole Aqp1b were cloned into the EcoRV/SpeI sites of the oocyte expression vector pT7Ts [63]. To obtain the Aqp1a-Ct1b, in which the C-terminus of Aqp1a was replaced by that of Aqp1b, the C-terminus-coding nucleotide sequence of Aqp1b was PCR amplified using a forward primer partially complementary to Aqp1a, 5'-CCCCCAAATTCCAAAACTTCAGGACGCGCAG-3', and a reverse primer bearing a SpeI restriction site, 5'-ACTAGTGCTTGTTTTTTCAGTGCTTTGG-3'. In parallel, a fragment of Aqp1a cDNA lacking the nucleotide sequence encoding the C-terminus was amplified using a forward primer specific to the 5' end of Aqp1a with an EcoRV site, 5'-GATATCGCCACCACCATGAGAGAG-3', and a reverse primer partially complementary to the nucleotide sequence of the C-terminus of Aqp1b, 5'-GAAGTTTTGGAATTTGGGGGACAG-3'. After purification, the two PCR products were used as templates to synthesize Aqp1a-Ct1b employing the forward and reverse primers bearing EcoRV and SpeI, respectively. The Aqp1b-Ct1a chimera, in which the C-terminus of Aqp1b was replaced by that of Aqp1a, was obtained by amplifying the C-terminus-coding nucleotide sequence of Aqp1a with the primers 5'-CACGAGCGGACGACTTCCCCGAGCGC-3' and 5'-ACTAGTCGTCTGTGTGGGACTATTTTGACG-3'. The Aqp1b-Ct1a cDNA was then amplified using the PCR product as reverse primer and a forward primer specific to the 5' end of Aqp1b, with an EcoRV site (5'-GATATCTCGACGCGGAGATGACAGAA-3'), employing full-length Aqp1b cDNA as a template. In all cases, PCR reactions were performed using Pwo or Easy-A high-fidelity polymerases (Stratagene), and the chimera cDNAs were ligated into pT7Ts after digestion with EcoRV and SpeI. Mutations into the sea bream Aqp1b C-terminal amino acid sequence were introduced by using the QuickChange site-directed mutagenesis kit (Stratagene) on pT7Ts-Aqp1b (Additional file 4). Sequence analysis of selected clones was carried out to confirm that only the desired chimeras or mutations were produced.

### Functional expression of teleost AQPs in *Xenopus laevis *oocytes

Complementary RNA (cRNA) synthesis, expression in *X. laevis *oocytes, and *P*_f _measurements were performed essentially as described [65]. Oocytes were injected with 0.25 to 10 ng cRNA. The swelling assays were carried out in 10-fold diluted modified Barth's solution (MBS: 88 mM NaCl, 1 mM KCl, 2.4 mM NaHCO_3_, 10 mM Hepes, pH 7.5, 0.82 mM MgSO_4_, 0.33 mM Ca(NO_3_)_2_, 0.41 mM CaCl_2_, and 25 μg/ml gentamicin) in the presence or absence of 0.7 mM HgCl_2 _and 5 mM β-mercaptoethanol. The data shown are an average of 3–5 experiments (each with different batches of oocytes), or from a representative experiment out of three different trials producing similar results. The measured *P*_f _values were statistically analyzed in an unpaired Student's *t *test; *p *values < 0.05 were considered significantly different.

### Western blotting and immunofluorescence microscopy

Total and plasma membranes were isolated from groups of 10 oocytes as described [66]. Protein samples were denatured at 95°C for 5 min in Laemmli buffer, electrophoresed on a 12% polyacrylamide SDS gel and then blotted onto PVDF or nitrocellulose membranes (Bio-Rad Laboratories). Membranes were blocked for 1 h with TBST (20 mM Tris, 140 mM NaCl, 0.1% Tween, pH 7.6) containing 1% nonfat dry milk, and then incubated with 1:300 diluted affinity-purified rabbit antisera against sea bream Aqp1a or Aqp1b in TBST with 1% nonfat milk powder at 4°C overnight. The Aqp1a and Aqp1b antisera were produced against synthetic peptides corresponding to the C-terminus of the corresponding proteins and they have been characterized elsewhere [10-12]. As secondary antibody, a 1:8000 dilution of goat anti-rabbit IgG coupled to horseradish peroxidase (Sigma) was used. Reactive protein bands were detected using enhanced chemiluminescence (Amersham). In some experiments, protein dephosphorylation was carried out before SDS-PAGE by resuspending total membrane extracts of Aqp1b-expressing oocytes in 10 mM MgCl_2_, 10 mM Tris-HCl, pH 7.5 and treating with calf intestinal alkaline phosphatase (Fermentas) for 6 h at 37°C, following the manufacturer's instructions.

Immunofluorescence microscopy was carried out on Aqp1a- and Aqp1b-expressing oocytes fixed in Bouin's without acetic acid for 4 h, subsequently dehydrated and embedded in paraplast (Sigma). Sections (7 μm) were blocked with 5% goat serum in PBST (137 mM NaCl, 2.7 mM KCl, 4.3 mM Na_2_HPO_4_, 1.4 mM KH_2_PO_4_, 1% BSA, 0.01% Tween, pH 7.5), and incubated at 4°C overnight with Aqp1a or Aqp1b antisera (1:100) in PBST with 1% goat serum. Bound antibodies were detected with mouse FITC anti-rabbit secondary antibodies (1:300). Sections were mounted with Vectashield (Vector Labs) or Prolong Gold antifade reagent (Invitrogen) and photos taken using a Leica SP2 confocal laser scanning microscope.

## Abbreviations

MIP: Membrane intrinsic protein; AQP: Aquaporin; FAAs: Free amino acids; Vtg: Vitellogenin; SaAQP1o: *Sparus aurata *aquaporin-1o (now termed Aqp1b); SaAQP1: *S. aurata *aquaporin-1 (now termed Aqp1a); EST: Expressed sequence tag; NJ: Neighbour-joining; ML: Maximum likelihood; BI: Bayesian inference; *P*_f_: Osmotic water permeability; CK1: Casein kinase 1; CK2: Casein kinase 2; WGD: Whole-genome duplication; MAPK: Mitogen-activated protein kinase; MPF: Maturation promoting factor.

## Authors' contributions

ATS carried out the cDNA cloning and the genomic and expression analyses, and the drafting of the manuscript. ATS, FC and MF performed the mutation experiments in oocytes and Western blotting. ATS and JL carried out the phylogenetic analyses, and DR was involved in immunocytochemical experiments. JC conceived and designed the study, contributed to coordination of tasks, and prepared the final version of the manuscript. All authors read and approved the final manuscript.

## Supplementary Material

Additional file 1 **Alignment of the amino acid sequences of vertebrate AQP1 and teleost Aqp1a and Aqp1b**. Alignment was performed using ClustalW employing the sequence from loop B to the start of the C-terminus, manually optimized using the Bioedit software. Conserved residues are shaded in black, residues conserved in at least 70% of the species in grey. Click here for file

Additional file 2 **Water permeability of *X. laevis *oocytes expressing sea bream wild-type (WT) or mutant Aqp1b**. The Aqp1b-S238A, Aqp1b-S253A, Aqp1b-S258A and Aqp1b-S262A mutants are shown. (A) Water permeability of oocytes expressing 1 ng cRNA of WT Aqp1b or the different mutants. Permeability is expressed in % related to oocytes injected with wild-type Aqp1b. Values represent the mean ± SEM of 3 experiments (each performed with different batches of oocytes; n = 10–15 oocytes per treatment). (B) Immunoblots of total membrane equivalents of oocytes expressing WT or mutant Aqp1b showing that all proteins were expressed at similar levels. The apparent molecular mass of a 29-kDa marker is indicated on the left. Click here for file

Additional file 3 **Degenerate and gene- or cDNA-specific oligonucleotide primers used for cloning and RT-PCR analysis**. The table list the oligonucleotide primers employed for the cloning of teleost AQP1-like cDNAs and sea bream *aqp1a* and *aqp1b* loci, and for RT-PCR analyses of *aqp1b *gene expression. Click here for file

Additional file 4 **Forward and reverse primers employed to introduce mutations into the sea bream Aqp1b cDNA**. The table lists the oligonucelotide primers employed for the site-directed mutagenesis of the sea bream Aqp1b cDNA. Click here for file
