# Supplementary material for: Structural and functional divergence of two fish aquaporin-1 water channels following teleost-specific gene duplication
Source: BMC Evol Biol. 2008 Sep 23;8:259. doi: 10.1186/1471-2148-8-259 (PMC2564943; doi:10.1186/1471-2148-8-259)
Supplement: Additional file 1 — Alignment of the amino acid sequences of vertebrate AQP1 and teleost Aqp1a and Aqp1b. Alignment was performed using ClustalW employing the sequence from loop B to the start of the C-terminus, manually optimized using the Bioedit software. Conserved residues are shaded in black, residues conserved in at least 70% of the species in grey. [file 1471-2148-8-259-S1.pdf]

Additional file 1

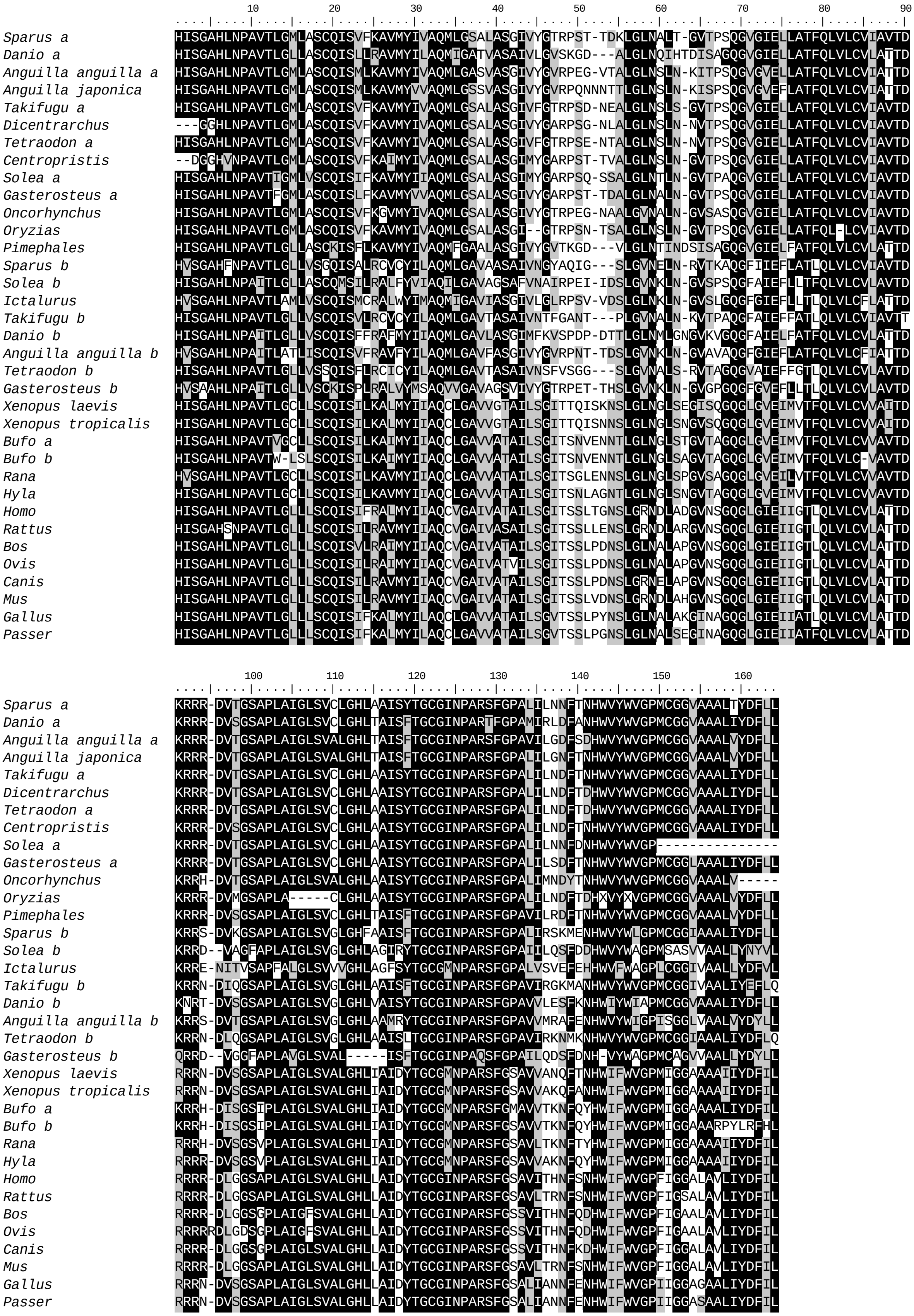

Alignment of the amino acid sequences of vertebrate AQP1 and teleost Aqp1a and Aqp1b. Alignment was performed using ClustalW employing the sequence from loop B to the start of the C-terminus, manually optimized using the Bioedit software. Conserved residues are shaded in black, residues conserved in at least 70% of the species in grey.
