# Supplementary material for: Structural and functional divergence of two fish aquaporin-1 water channels following teleost-specific gene duplication
Source: BMC Evol Biol. 2008 Sep 23;8:259. doi: 10.1186/1471-2148-8-259 (PMC2564943; doi:10.1186/1471-2148-8-259)
Supplement: Additional file 2 — Water permeability of X. laevis oocytes expressing sea bream wild-type (WT) or mutant Aqp1b. The Aqp1b-S238A, Aqp1b-S253A, Aqp1b-S258A and Aqp1b-S262A mutants are shown. (A) Water permeability of oocytes expressing 1 ng cRNA of WT Aqp1b or the different mutants. Permeability is expressed in % related to oocytes injected with wild-type Aqp1b. Values represent the mean ± SEM of 3 experiments (each performed with different batches of oocytes; n = 10–15 oocytes per treatment). (B) Immunoblots of total membrane equivalents of oocytes expressing WT or mutant Aqp1b showing that all proteins were expressed at similar levels. The apparent molecular mass of a 29-kDa marker is indicated on the left. [file 1471-2148-8-259-S2.pdf]

Additional file 2

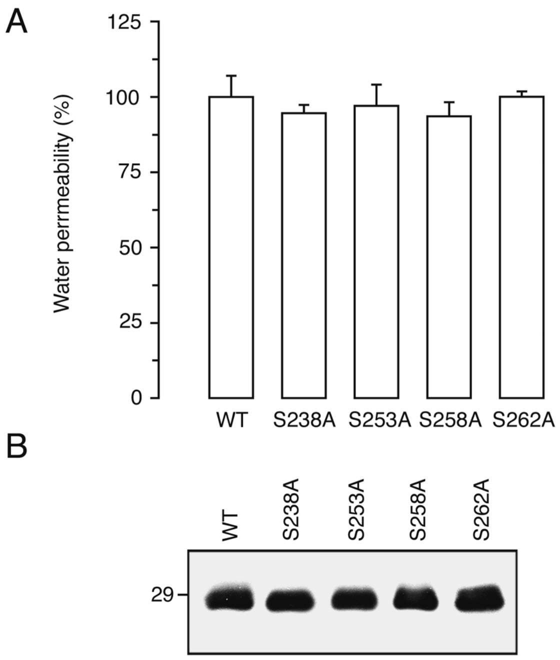

Water permeability of *X. laevis* oocytes expressing sea bream wild-type (WT) or mutant Aqp1b. The Aqp1b-S238A, Aqp1b-S253A, Aqp1b-S258A and Aqp1b-S262A mutants are shown. *A*, water permeability of oocytes expressing 1 ng cRNA of WT Aqp1b or the different mutants. Permeability is expressed in % related to oocytes injected with wild-type Aqp1b. Values represent the mean ± SEM of 3 experiments (each performed with different batches of oocytes; *n* = 10-15 oocytes per treatment). *B*, immunoblots of total membrane equivalents of oocytes expressing WT or mutant Aqp1b showing that all proteins were expressed at similar levels. The apparent molecular mass of a 29-kDa marker is indicated on the left.
