# Supplementary material for: Structural and functional divergence of two fish aquaporin-1 water channels following teleost-specific gene duplication
Source: BMC Evol Biol. 2008 Sep 23;8:259. doi: 10.1186/1471-2148-8-259 (PMC2564943; doi:10.1186/1471-2148-8-259)
Supplement: Additional file 3 — Degenerate and gene- or cDNA-specific oligonucleotide primers used for cloning and RT-PCR analysis. The table list the oligonucleotide primers employed for the cloning of teleost AQP1-like cDNAs and sea bream aqp1a and aqp1b loci, and for RT-PCR analyses of aqp1b gene expression. [file 1471-2148-8-259-S3.pdf]

Additional file 3

Degenerate and gene- or cDNA-specific oligonucleotide primers used for cloning and RT-PCR analysis

Gene	Reference/ GenBank No.	Forward primer 5'-3'/ Reverse primer 5'-3'	Purpose
MIP family-related genes	[1]	GGNGSNCAYNTNAAYCCNGC/ GGDSCVARNSWBCKNGCNGG	Degenerate primers for AQP cDNA cloning
aqp1a	This work	GACCAGGARRTSAAGGTG/ CACATVGGCCCSACCCAG	Teleost Aqp1b cDNA cloning
Sparus aurata aqp1a	AY626939	CCCACAGCCAGACAGCAACA/ GTCTGTGTGGGACTATTTTGACG	Cloning of *aqp1a* gene
S. aurata aqp1b	AY626938	TCGACGCGGAGATGACAGAA/ TCAGCCATATTGAAAGCTTTTCTGC	Cloning of *aqp1b* gene
S. aurata aqp1b	AY626938	GCGACGGAGTGATGTCAAAGG/ AGATAAGAGCCGCCGCTATGC	RT-PCR expression analysis
S. aurata aqp1a/aqp1b	AY626939, AY626938	CGACGTTAACGGAGGCAATG/ CCGATGATGGCGGTCAAAC	Amplification of *aqp1a* and *aqp1b* flanking cassette
Anguilla anguilla aqp1b	EF011738	CATTCTGGCGCAGATGCTTG/ TTTGGGGTGGAGGAGGTAGTCA	RT-PCR expression analysis
Solea senegalensis aqp1b	AY626941	TGCCAGCTGCCAGATGAGTATC/ AACATGCTCCAGCAGAGGTTCC	RT-PCR expression analysis
Danio rerio aqp1b	XM_682613	ACTGTAGTAATGGCACGAGAGC/ TTTACAAGAGGCATTTCAGGG	Cloning of Aqp1b cDNA
D. rerio aqp1b	EU327345	GCCACCTTTCAGCTGGTTCTGT/ ATGGGGGCAATCCAGTAAATCC	RT-PCR expression analysis
bactin1	NM_131031	ACATGGAGAAGATCTGGC/ GCGTACAGGTCCTTACGGA	RT-PCR expression analysis

1. Preston GM: **Cloning of gene family members using the polymerase chain reaction with degenerate oligonucleotide primers.** *Methods Mol Biol* 1997, **69**:97-113.
