# Supplementary material for: Structural and functional divergence of two fish aquaporin-1 water channels following teleost-specific gene duplication
Source: BMC Evol Biol. 2008 Sep 23;8:259. doi: 10.1186/1471-2148-8-259 (PMC2564943; doi:10.1186/1471-2148-8-259)
Supplement: Additional file 4 — Forward and reverse primers employed to introduce mutations into the sea bream Aqp1b cDNA. The table lists the oligonucelotide primers employed for the site-directed mutagenesis of the sea bream Aqp1b cDNA. [file 1471-2148-8-259-S4.pdf]

Additional file 4

Forward and reverse primers employed to introduce mutations into the sea bream Aqp1b cDNA

	Forward primer 5'-3'/Reverse primer 5'-3'
T229A	CACGAGCGCAAAACTTCAGGGCGCGCAGGAATGTTCTGC/<br>GCAGAACATTCCTGCGCGCCCTGAAGTTTTGCGCTCGTG
T229D	GCGCAAAACTTCAGGGACCGCAGGAATGTTCTG/<br>CAGAACATTCCTGCGGTCCCTGAAGTTTTGCGC
L234A/L235A	ACGCGCCGGAATGTTGCGGCTAATGGTTCAGAA/<br>TTCTGAACCATTAGCCGCAACATTCCGGCGCGT
S238A	GGAATGTTCTGCTTAATGGTGCAGAAGATGAAGACGCTGG/<br>CCAGCGTCTTCATCTTCTGCACCATTAAGCAGAACATTCC
S253A	GACGCACCTAGAGAAGGCAACGCCAGCCCGGGGCCAAGTCAGGG/<br>CCCTGACTTGGCCCCGGGCTGGCGTTGCCTTCTCTAGGTGCGTC
S254A	CTAGAGAAGGCAACAGCGCCCCGGGGCCAAGTCAG/<br>CTGACTTGGCCCCGGGGCGCTGTTGCCTTCTCTAG
S254D	CTAGAGAAGGCAACAGCGACCCGGGGCCAAGTCAG/<br>CTGACTTGGCCCCGGGTCGCTGTTGCCTTCTCTAG
S258A	GCAACAGCAGCCCGGGGCCAGCTCAGGGGCCAAGTCAGTGGC/<br>GCCACTGACTTGGCCCCTGAGCTGGCCCCGGGCTGCTGTTGC
S262A	CCGGGGCCAAGTCAGGGGCCAGCTCAGTGGCCAAAGCACTG/<br>CAGTGCTTTGGCCACTGAGCTGGCCCCTGACTTGGCCCCGG

Mutated codons are underlined.
